# Development of a deep learning-based tool for coronary artery stenosis evaluation in forensic autopsies using whole slide imaging

**DOI:** 10.1007/s00414-026-03800-6

**Published:** 2026-04-18

**Authors:** Nicola Pigaiani, Shakiba Sharifi, Marianna Garavello, Francesco Setti, Francesco Ausania, Federica Bortolotti, Alberto Chighine, Ernesto D’Aloja, Pamela Rodegher, Serena Ammendola, Matteo Brunelli, Marco Cristani, Stefano Gobbo

**Affiliations:** 1 Section of Forensic Medicine, Department of Diagnostics and Public Health, University of Verona, P.le L.A. Scuro 10, Verona, Italy; 2 Section of Engineering and Physics, Department of Engineering for Innovation Medicine, University of Verona, Verona, Italy; 3 Section of Legal Medicine, Department of Medical Science and Public Health, University of Cagliari, Cagliari, Italy; 4 Unit of Pathology, Azienda Ospedaliera Universitaria Integrata, Verona, Italy; 5 Section of Pathology, Department of Diagnostics and Public Health, University of Verona, Verona, Italy

**Keywords:** Artificial intelligence, Digital pathology, Whole slide images, Coronary stenosis, Cardiovascular pathology, Forensic pathology

## Abstract

**Background:**

Cardiovascular disease is a leading cause of mortality, with coronary artery disease accounting for over 60% of cases in adults. Accurate quantification of coronary stenosis in forensic autopsies is crucial for determining causality between pathological findings and death, but is hindered by subjective visual assessments and inter-observer variability. This study aimed to develop an AI-driven tool using whole-slide images for objective stenosis measurement in forensic investigations.

**Materials and methods:**

From 98 anonymized H&E-stained autopsy slides (234 coronary sections), 103 high-quality regions of interest were selected and split into training (*n* = 82), validation (*n* = 14), and test (*n* = 7) datasets. Annotations delineated lumen, internal elastic lamina, and external elastic lamina using QuPath. A SegFormer-B0 transformer model was trained with data augmentation, weighted cross-entropy loss, and AdamW optimization. Post-processing enforced anatomical structural hierarchy and generated hybrid confidence maps.

**Results:**

On validation dataset, agreement with ground truth was excellent (MAE 3.22% points; RMSE 4.00; MAPE 6.07%; *r* = 0.986; ICC = 0.986), with slight underestimation. Bland–Altman bias was − 1.36 pp (95% LoA − 9.01 to 6.28 pp), indicating performance across severities. On the test set, accuracy improved (MAE 1.01 pp; RMSE 1.50; MAPE 2.11%; *r* = 0.998; ICC = 0.995) and outperformed three pathologists’ visual estimates (MAE 16.11, 8.18, 4.79 pp). Bland–Altman bias for the model was − 0.82 pp with tight limits of agreement (− 3.48 to 1.83 pp). Total inference time was 199.08 s for seven cases (28.44 s/image).

**Conclusions:**

WSI-based transformer pipeline enables rapid, auditable, and reproducible coronary stenosis measurement, reducing inter-observer variability and supporting standardized interpretation in forensic investigations.

**Supplementary Information:**

The online version contains supplementary material available at 10.1007/s00414-026-03800-6.

## Introduction

Determining the cause of death is a core purpose of forensic autopsies. As sudden and unexpected deaths typically fall within the remit of medico-legal investigation, cardiovascular deaths frequently come to the attention of forensic pathologists rather than being investigated solely from the clinical perspective.

In sudden cardiac deaths (SCDs), a subgroup of cardiovascular deaths, coronary atherosclerosis accounts for more than 60% of cases in adults, making sudden death due to advanced coronary atherosclerosis and critical stenosis a central target for forensic post-mortem investigation. In this context, the thorough examination of the epicardial coronary arteries and their major branches is a critical phase of forensic autopsy [1].

It is well-known that within SCD due to coronary stenosis, macroscopic and microscopic signs vary depending on the survival time following the precipitating event. The earliest myocardial histological changes, such as wavy fibers, early coagulative necrosis, edema, hemorrhage, and cytoplasmic hypereosinophilia, may become appreciable after ~ 4–12 h [2]. If the death occurs before this time window, documenting an acute occlusion or a critical stenosis can offer the most compelling anatomical support for an ischemic mechanism. In this context, considering the well-established association between acute ischemia and the onset of malignant ventricular tachyarrhythmias, assessing hemodynamically significant coronary stenosis in cases of sudden unexplained death may be pivotal to the final interpretation [3, 4].

Based on this, the evaluation of the coronary arteries during autopsy should proceed with meticulous scrutiny and adherence to standardized protocols to ensure accurate and reliable findings: this generally entails serial transverse sections at 3-mm intervals along the course of main epicardial coronary artery trunks to map plaque distribution and any thrombotic material and to select the most critical segments for histological sampling in order to microscopically characterize lesion type and morphology (e.g., plaque complications and thrombosis) [5].

Quantifying the degree of stenosis is relevant because widely used thresholds define when a lesion is likely to be hemodynamically significant (≥ 70% luminal reduction) [6]. Moreover, in autopsy-oriented approaches to sudden cardiac death, a stenosis of 75% or greater may be considered sufficient evidence to support a highly probable coronary cause, particularly when coronary pathology represents the only objective finding [5].

Despite its significance, numerous studies have demonstrated that the grading of coronary stenosis remains vulnerable to operator dependence and inter-observer variability. More importantly, training and experience alone are not invariably sufficient to ensure accurate assessment of the degree of stenosis [7, 8].

To mitigate subjective interpretation and enhance consistency in evaluating luminal stenosis, Barth et al. developed a visual guide by selecting representative transverse coronary sections, using Adobe Photoshop’s scaling and measurement tools to produce standardized stenosis templates [9]. However, a notable limitation of this visual guide is its dependence on discretized stenosis categories, spaced by 20% increments for mild narrowing and 10% increments for severe stenosis, which may not adequately represent the continuous spectrum of real-world lesions (≈ 20%, 40%, 60%, 70%, 80%, and 90% luminal narrowing).

Advancing beyond discretized visual templates, the recent progression of digital pathology via whole-slide images (WSIs) has resulted in high-resolution, archivable, and easily shareable digital replicas of histological slides that facilitate examination through image analysis tools, enabling objective and reproducible quantification of morphologic patterns that would otherwise be prone to inter-observer variability [10–14]. In cases of SCDs attributable to coronary stenosis, digital pathology could provide an unbiased, image-based framework for continuous quantification of luminal narrowing, rather than relying on fixed visual categories [15].

The aim of this research was to develop and validate an artificial intelligence (AI)-driven tool utilizing WSIs to enhance the accuracy and consistency of coronary stenosis quantification taken from real-life medico-legal cases collected in two different Forensic Institutes. This approach aims to reduce interobserver variability while streamlining the process, thus holding the potential to establish a new reproducible diagnostic tool.

## Materials and methods

### Records selection

A total of 98 anonymized hematoxylin and eosin (H&E)-stained glass slides, each from a different decedent, were extracted from the forensic autopsy databases of two Forensic Medicine Institutes. The slides were prepared from paraffin-embedded tissue blocks containing autopsy-derived coronary artery cross-sections with varying degrees of stenosis, sampled in accordance with cardiovascular pathology guidelines, decalcified, and processed by certified histology laboratories.

Each slide contained 1 to 4 arteries, for a total of 234 sections. The whole dataset encompassed segments that represented all branches of the coronary tree (right coronary, left main coronary, left circumflex, and left anterior descending artery). Each coronary section was considered a region of interest (ROI).

All glass slides were scanned using the GT 450 DX Digital Slides Scanner (Leica Biosystems, Nussloch, Germany), and acquired at 40× magnification; time of scanning ranged between 1.5 and 2.3 min for each slide. Digital files, sized from 770.5 Mb to 2,260 Mb, were anonymized by assigning a progressive identifier to each file and uploaded to a local server with restricted access to authorized personnel for storage and image retrieval.

Two forensic pathologists, experts in digital pathology, used a designated digital viewer (QuPath version 0.6.0) [16] running on an ultra-high-definition (8 K) monitor with a 120 Hz refresh rate to select 103 high-quality ROIs, excluding ROIs affected by artifacts such as dust, air bubbles, or scanner-related light scattering. ROIs, namely a single artery section, were randomly split into training (*n* = 82), validation (*n* = 14), and test (*n* = 7) datasets (Table 1).

**Table 1:** WSIs splitting, purposes, and technical execution of each step

Set name (ROIs)	Purposes	Technical execution
Training Set (*n* = 82)	The model learns to identify coronary anatomy from expert-labeled annotations.	Internal 70/30 split of the 82 ROIs used for model parameter optimization and weight tuning.
Validation Set (*n* = 14)	Testing the model’s accuracy on “unseen” independent slides to calculate objective error rates.	Direct comparison between model predictions and manual QuPath ground truth area measurements.
Test Set (*n* = 7)	Evaluating the tool’s performance against visual judgment of three human experts.	Assessment of inter-observer variability and tool reliability in a real-life setting.

### Annotation step

**Fig. 1:**
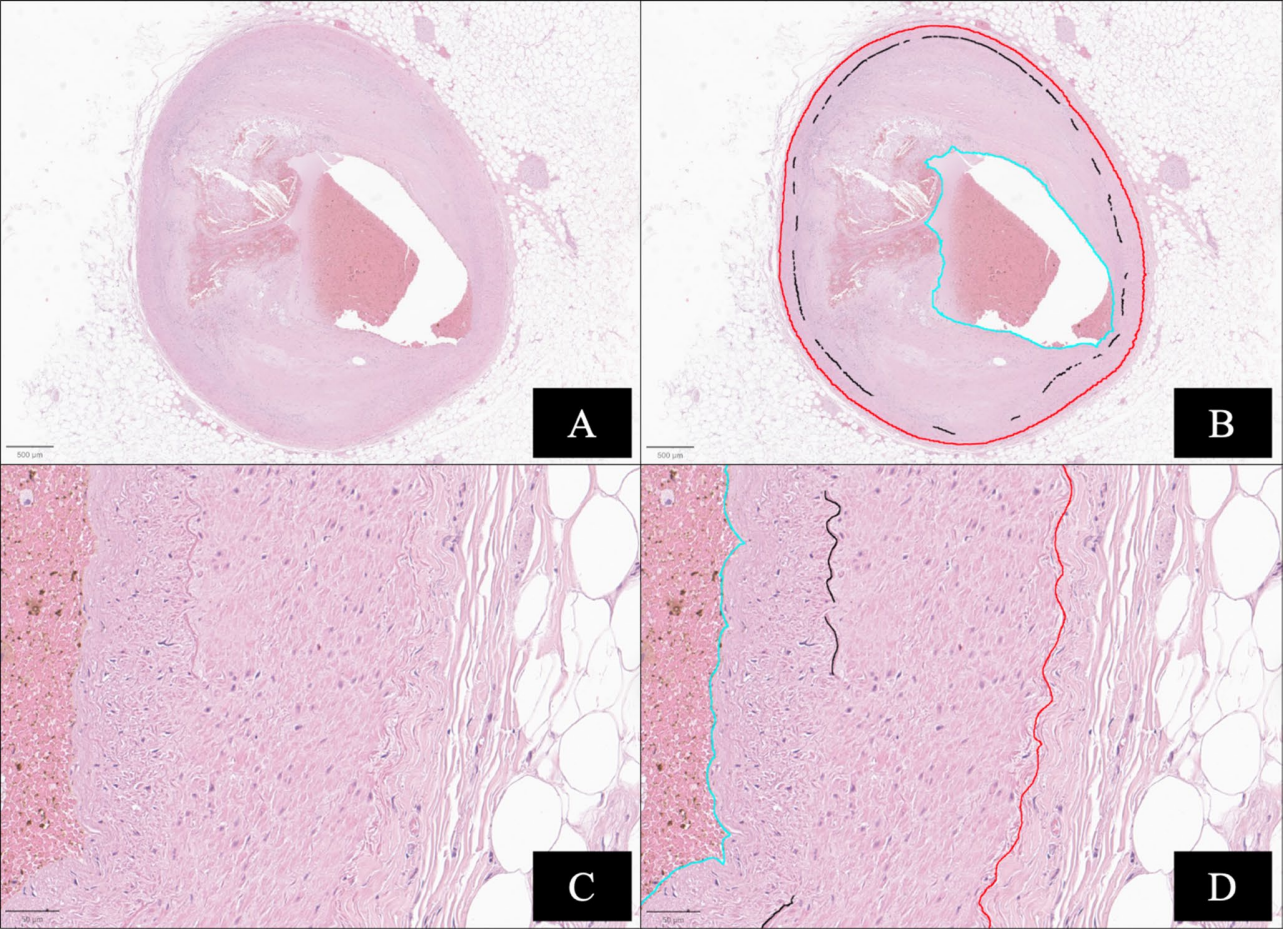
Example of the annotating process of a coronary with actual stenosis of 74.15%. Blue: lumen; black: internal elastic lamina (IEL), note the discontinuous appearance of IEL due to the sectioning artifacts; red: external elastic lamina (EEL). **A**: ROI without annotations (WSI, H&E, 2X magnification); **B**: ROI with complete annotations (WSI, H&E, 2X magnification); **C**: detail of arterial wall without annotations (WSI, H&E, 20X magnification); **D**: detail of arterial wall with annotations (WSI, H&E, 20X magnification)

After retrieving images from the server, 49 WSIs containing 82 ROIs of the training dataset were uploaded to QuPath version 0.6.0. A trained forensic pathologist, following the European Society of Digital and Integrative Pathology (ESDIP) recommendations [17] conducted annotations using an NP754QHA-KA1IT Galaxy Book5 notebook and a digital pen (Samsung Electronics Co., LTD, Dublin, IE) for the training process. In particular the pathologist outlined the following objective structures (Fig. 1):

- the residual *lumen* area: residual area of the *lumen* still patent;
- the internal elastic lamina (IEL): the discontinuous layer composed of a condensed, wavy, and corrugated sheet of elastic fibers forming the boundary between the *tunica intima* and *tunica media* (i.e., the presumed original lumen edge [1]);
- the external elastic lamina (EEL), the layer separating the *tunica media* from the *tunica adventitia*.

The labelling was performed at high magnification (30x), in order to maximize morphological accuracy.

All annotations were verified by a second skilled forensic pathologist, saved as a “.*geojson*” file (a JSON-based format used to store and exchange spatially referenced geometric data and associated attributes) [18], and uploaded to the restricted-access server.

### Training dataset

#### Data preparation

The initial phase of the technical workflow involved the engineering of raw forensic data to ensure high-fidelity spatial and anatomical accuracy. A total of 82 high-quality ROIs were systematically extracted from the forensic autopsy database, representing a diverse range of coronary artery sections. Using a custom Python script, implementing a rule-based cropping logic, each artery was isolated from the WSI while maintaining a standardized 1500-pixel background margin. This margin was mathematically essential for transformer-based models as it provided contextual spatial information that allowed the system to accurately differentiate between the EEL and the surrounding perivascular tissue, a critical capability when analyzing the ruined or fragmented tissue often encountered in forensic autopsies [19]. Following extraction, global WSI coordinates were transformed into local ROI coordinates and resized to a consistent 1024 × 1024 resolution. To address the challenges posed by a small-sample forensic dataset, the *albumentations* Python library was used to perform data augmentation [20]. The process did not increase the number of samples, which remained constant at 82 original ROIs. During the training phase, the model underwent 200 learning cycles, thereby exposing it to thousands of unique variations of the original 82 cases. In each cycle, the system was presented with a different perspective on each sample, generated by applying random, temporary transformations to the original images. Geometric transformations, such as rotations and elastic deformations, were employed to simulate physical tissue warping and varying slide orientations, while color augmentations, including hue and saturation shifts, accounted for the inherent variability in H&E staining intensity across different laboratory environments. This methodology ensures that the system learns to recognize anatomical landmarks based on structural truths rather than memorizing the specific appearance of static images, thereby enhancing its robustness in handling fragmented or variably stained tissues commonly encountered in real-world forensic autopsies.

#### Training process

Model training was performed using a supervised learning approach. The system was built upon the SegFormer-B0 architecture, a state-of-the-art transformer-based model [21]. Unlike traditional AI frameworks that process images through localized pixel filters, this architecture employs self-attention mechanisms that allow the model to evaluate the entire 1024 × 1024 field of view simultaneously. This global awareness is vital for forensic pathology applications because it enables the AI to recognize the circular continuity of an artery even if the structural boundaries are partially degraded or broken.

The model was specifically trained to identify a 4-class hierarchical segmentation strategy, including background (0) (i.e., the space outside the EEL), *tunica media* (1) (i.e., the region between EEL and IEL), *tunica intima* (2) (i.e., the region between the IEL and the edge of the residual lumen), and *lumen* (3).

To ensure the model did not overlook the tunica intima in favor of the larger background area, a weighted Cross-Entropy Loss function was implemented [22]. This mathematical weighting strategy assigned higher importance to pixels associated with the thinner arterial layers, calculated inversely to their frequency in the dataset using the formula:

$$\:{W}_{class=\:\frac{Total\:Pixels}{4\:*\:Class\:Counts+\:{10}^{-8}}}$$

Optimization was managed via the *AdamW* optimizer [23]. This allowed the core transformer backbone to learn at a more conservative speed (1 * 10^− 5^) to preserve foundational features, while the classifier head was tuned at a faster rate (1 * 10^− 4^) to adapt specifically to the unique textures and morphologies of forensic coronary sections.

**Fig. 2:**
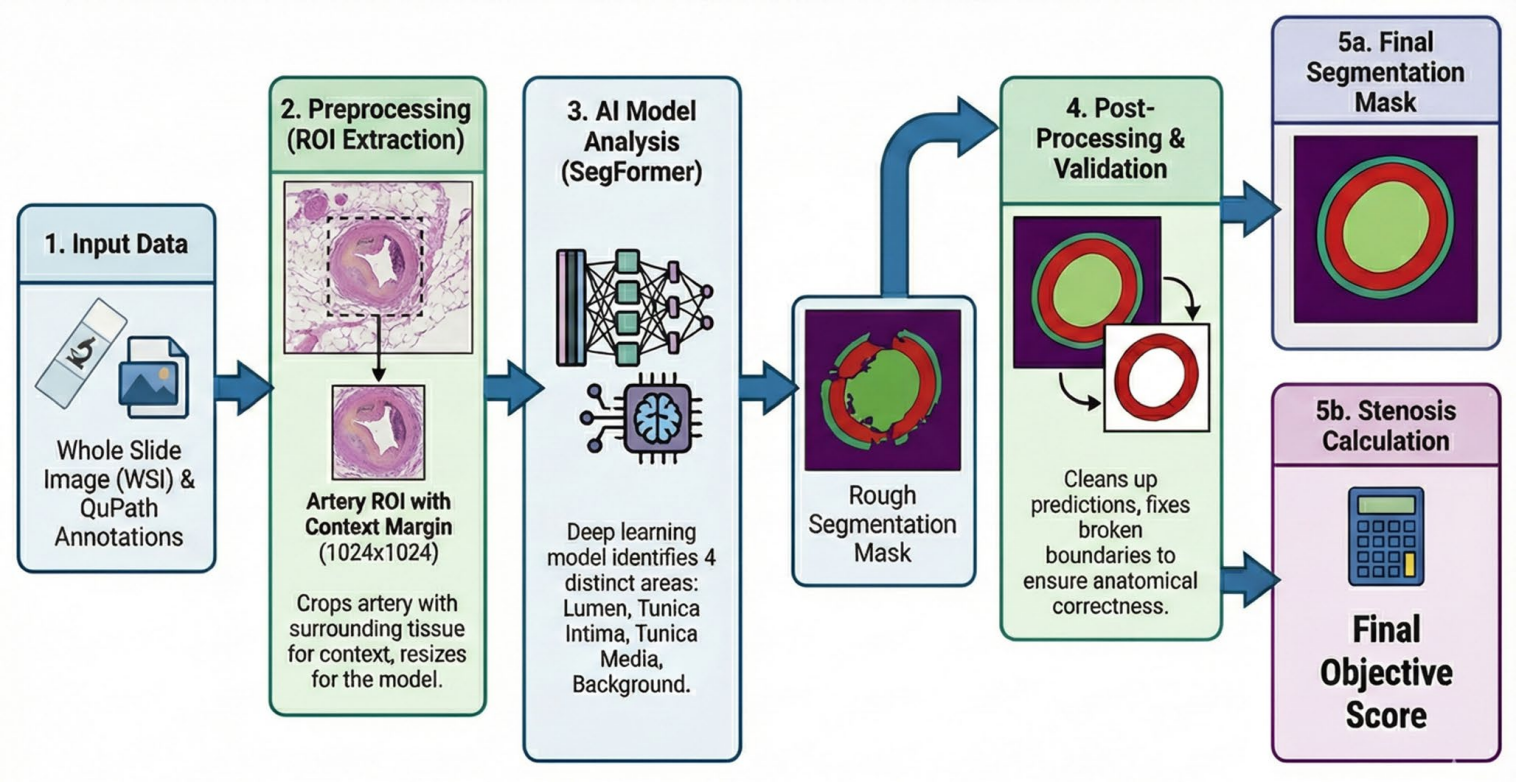
Simplified workflow of the Deep Learning tool from raw autopsy WSIs to objective stenosis percentage

The stability of the training was monitored by a scheduler to reduce the learning rate upon plateau occurrence, which moderated the learning speed if progress stalled. Furthermore, an early stopping protocol with a patience of 25 epochs was enforced to prevent overfitting, a technical state where the AI memorizes specific training images instead of learning the generalized anatomical rules necessary for reliable forensic diagnostic use (Fig. 2).

### Validation dataset

**Fig. 3:**
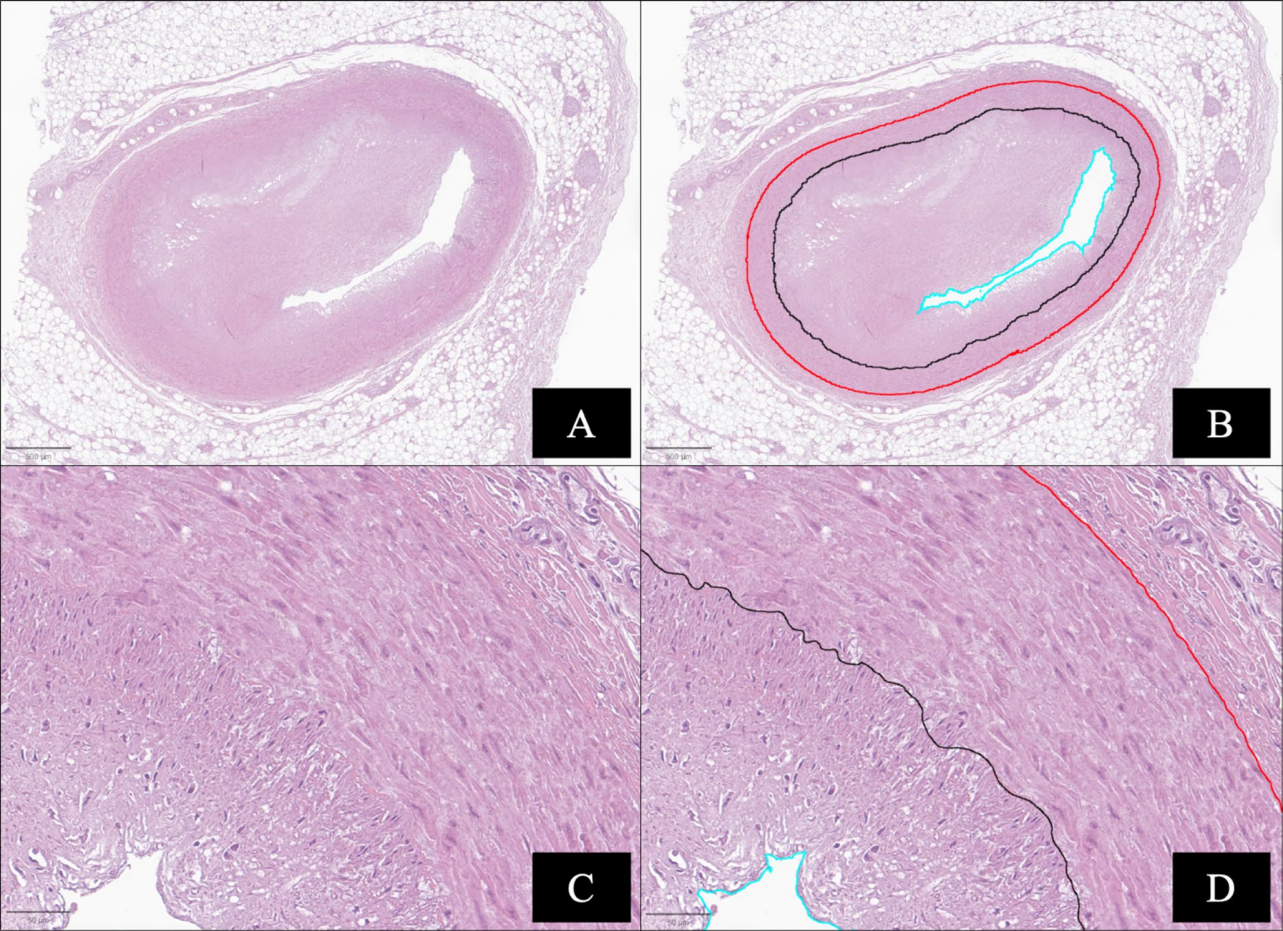
Example of ground truth definition in a coronary with actual stenosis of 93.96%. Blue: lumen; black: internal elastic lamina, note the continuous appearance of IEL due to interpolation gaps based on the discernible intima–media interface; red: external elastic lamina. **A**: ROI without annotations (WSI, H&E, 2X magnification); **B**: ROI with complete annotations (WSI, H&E, 2X magnification); **C**: detail of arterial wall without annotations (WSI, H&E, 20X magnification); **D**: detail of arterial wall with annotations (WSI, H&E, 20X magnification)

Upon retrieving images from the server, 7 WSIs of the validation dataset, containing 14 ROIs, were uploaded to QuPath version 0.6.0. Annotations were performed as previously described (see *Annotation step*). In the validation set, differently from the training set, the internal elastic lamina (IEL) was annotated as a continuous contour interpolating across gaps where it was not clearly visible, based on the discernible intima–media interface (Fig. 3) to determine the ground truth.

Ground truth was delineated by a forensic pathologist based on the annotations, calculating the exact stenosis percentages (S_p_) using the area measurement function available in QuPath version 0.6.0 and applying the formula:

$$\:{S}_{p}=\left[1-\left(\frac{Residual\:Lumen\:\left({\mathrm{m}}^{2}\right)}{Residual\:Lumen\:\left({\mathrm{m}}^{2}\right)+Tunica\:intima\:\left({\mathrm{m}}^{2}\right)}\right)\right]\:*100$$

For the validation, annotations used for the ground truth were not provided to the model, which evaluated the same, previously unseen, 14 unlabeled ROIs without annotations.

Once the trained model generated its initial prediction of artery in raw pixel masks, a sequential anatomical post-processing pipeline was implemented to ensure the results remained consistent with biological reality. To enhance the roughly produced masks and fragmented boundaries, the system applied a Convex Hull Constraint, which mathematically wraps the artery to eliminate unrelated background noise or far-field false positives [24]. Furthermore, an inner ring reconstruction step was utilized to fill gaps in fragmented sections of the tunica intima. This process followed strict anatomical hierarchy rules, ensuring that the lumen was always nested within the inner class (tunica intima), which is itself contained within the outer class (tunica media). To preserve the integrity of the AI’s primary findings, these corrective modifications were only applied to pixels where the model’s confidence score fell below a threshold of 0.65.

To address the challenges posed by ruined or fragmented prediction, the system generates a hybrid confidence map for every diagnosis. This map serves as a transparency layer and enhances clinical safety by providing a transparent audit trail of the AI’s diagnostic certainty. Unlike traditional models, this system accounts for uncertainty introduced during post-processing.

The system identifies pixels modified by these interventions such as filling gaps in the tunica intima or tunica media and applies a 0.7 penalty factor, reducing their confidence score by 30%.

The final quantitative output, the stenosis percentage (S_p_), was calculated by the model comparing the surface areas (number of pixels) of the predicted masks using the same formula used for the ground truth.

This AI-derived continuous percentage was then compared directly with the expert’s manual ground truth area measurements in QuPath. The accuracy of the tool was formally quantified using the Percentage Error (%Er) formula:

$$\:\%Er=\frac{(Model\:Stenosis\:Predicion-Ground\:Truth)}{Ground\:Truth}*100$$

This rigorous comparison allowed for a precise evaluation of the tool’s performance across the full spectrum of coronary narrowing, from mild atherosclerosis to critical stenosis.

### Test dataset

The final evaluation phase used the Test Dataset, comprising 7 unlabeled high-quality ROIs without annotations, to benchmark the AI tool’s performance against the visual judgments, without metrical tools, of experienced pathologists.

In the test set, a senior forensic pathologist delineated the ground truth at 30x magnification and calculated the exact stenosis percentages (S_p_) using the area measurement function available in QuPath version 0.6.0, based on the annotations performed as described for the validation dataset (i.e., IEL annotated as a continuous conformation; Fig. 3).

During this phase, three senior forensic pathologists, unaware of S_p_, independently performed visual evaluations of each unlabeled ROI in QuPath (version 0.6.0) to assess the percentage of coronary stenosis.

To facilitate a direct comparison, the AI model was deployed in a specialized minimal inference mode which optimized computational resources by focusing exclusively on the core 4-class segmentation and area-based calculations while disabling auxiliary visualizations. This allowed the tool to process each coronary section at a consistent 1024 × 1024 resolution, ensuring that every pixel was accounted for in the final quantification.

A hybrid confidence map for every diagnosis as reported above was generated by the system, also for the test dataset. This approach allows the final stenosis percentage (S_p_) to be presented with a realistic estimate of reliability.

Finally, model estimations and forensic pathologists’ visual assessment were statistically compared with the ground truth.

### Statistics

All statistical analyses were performed using R (R Foundation for Statistical Computing, Vienna, Austria), and graphical figures were generated and exported using jamovi (The jamovi project). Stenosis percentage (S_p_) values were treated as continuous variables. For each ROI, the absolute error was computed as predicted stenosis – ground truth reported in percentage points (pp) and the relative error as percent error. Model performance was quantified using mean absolute percentage error (MAPE), mean absolute error (MAE), and root mean square error (RMSE) on the absolute scale (pp). For percent errors, variability was described using the sample standard deviation (SD) and the 95% confidence interval (CI) of the mean was calculated with the Student’s t distribution (two-sided, df = *n*−1). Pearson’s correlation (two-tailed) was used to assess linear association, with 95% CIs derived by Fisher’s z transformation. Agreement was evaluated using Bland–Altman bias and 95% limits of agreement and by the intraclass correlation coefficient (ICC, two-way random-effects, absolute agreement, single measure). For Bland–Altman and ICC estimates, robust 95% confidence intervals were obtained via nonparametric bootstrap (10,000 resamples of ROIs).

## Results

### Validation dataset results

The validation set consisted of 14 ROIs. These ROIs were processed by the model to generate quantitative stenosis percentages, which were then compared to the ground truth established by a forensic pathologist using QuPath’s manual area measurement tools.

**Fig. 4:**
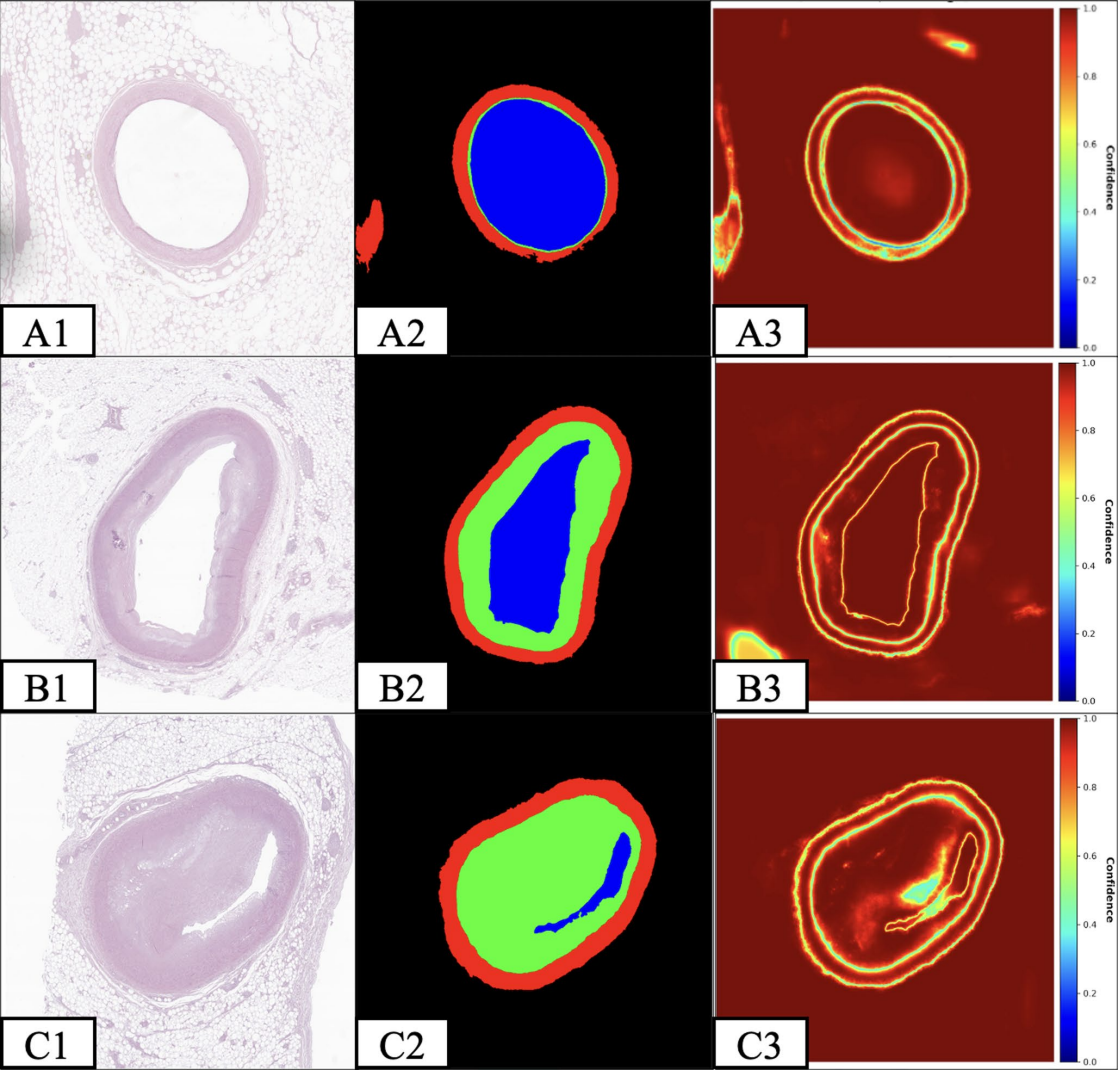
**A**, **B**, **C** (VD-1 – actual stenosis 6.12%, model predicted stenosis 5.72%; VD-5 – actual stenosis 55.42%, model predicted stenosis 54.87%; VD-6 samples – actual stenosis 93.96%, model predicted stenosis 92.71%): three examples of model stenosis prediction. A1, B1, C1: raw ROIs (H&E, 2x magnification). A2, B2, C2: model masks predictions (black: background; red: tunica media; green: tunica intima; blue: lumen). A3, B3, C3: confidence heat maps of predictions

Results are shown in Table sm1 – supplementary material. The model performance is shown in Table 2. Figure 4 shows the model segmentation and heat confidence map.

**Table 2:** Model performances on validation set

Metric	Value	Unit
Mean percent error	−2.87	%
Standard deviation of percent error	7.20	%
95% CI of the mean percent error	[− 7.02; +1.29]	%
Mean bias (predicted stenosis – GT)	−1.36	pp
MAPE	6.07	%
MAE	3.22	pp
RMSE	4.00	pp
Pearson correlation r (GT vs. Model prediction)	0.9864	
p (two-tailed)	*p* < 0.0001	
95% CI for r	[0.9564; 0.9958]	
Bland–Altman bias	−1.36	pp
95% LoA	[− 9.01, 6.28]	pp
Bias 95% CI (boot)	[− 3.23, 0.77]	pp
LoA low 95% CI (boot)	[− 11.35, − 5.63]	pp
LoA high 95% CI (boot)	[1.58, 10.41]	pp
ICC	0.9856	
ICC 95% CI (boot)	[0.9342, 0.9956]	

*CI* confidence interval, *GT* ground truth, *pp* percentage points, *MAPE* mean absolute percentage error, *MAE* mean absolute error, *RMSE* root mean square error, *LoA* level of agreement, *ICC* intraclass correlation coefficient, *pp* percentage points

Across the 14 ROIs, the model showed a slight overall underestimation of stenosis. The average magnitude of error was MAE 3.22 pp with RMSE 4.00 pp. MAPE was 6.07%. On the relative scale, the mean percent error was − 2.87% with a sample SD of 7.20% and a 95% CI for the mean of − 7.02% to + 1.29%. Ground truth and model predictions were strongly associated (Pearson’s *r* = 0.9864, two-tailed *p* < 0.001; 95% CI = 0.9564–0.9958). Bland–Altman analysis showed that the model slightly underestimated stenosis relative to ground truth, with a mean bias of − 1.36 pp (bootstrap 95% CI − 3.23 to 0.77). The 95% limits of agreement ranged from − 9.01 to + 6.28 pp ( ≈ ± 8 pp), indicating that most model–reference differences fell within this interval. Consistently, the ICC for absolute agreement was 0.986 with a bootstrap 95% CI of 0.935–0.996, supporting excellent agreement between model predictions and the ground-truth measurements across the full set of ROIs.

### Test dataset results

The final 7 ROIs served as the blind test. The model required 199.08 s to analyze and process the whole dataset (seven coronary stenosis estimates), corresponding to a mean processing time of 28.44 s per image.

**Fig. 5:**
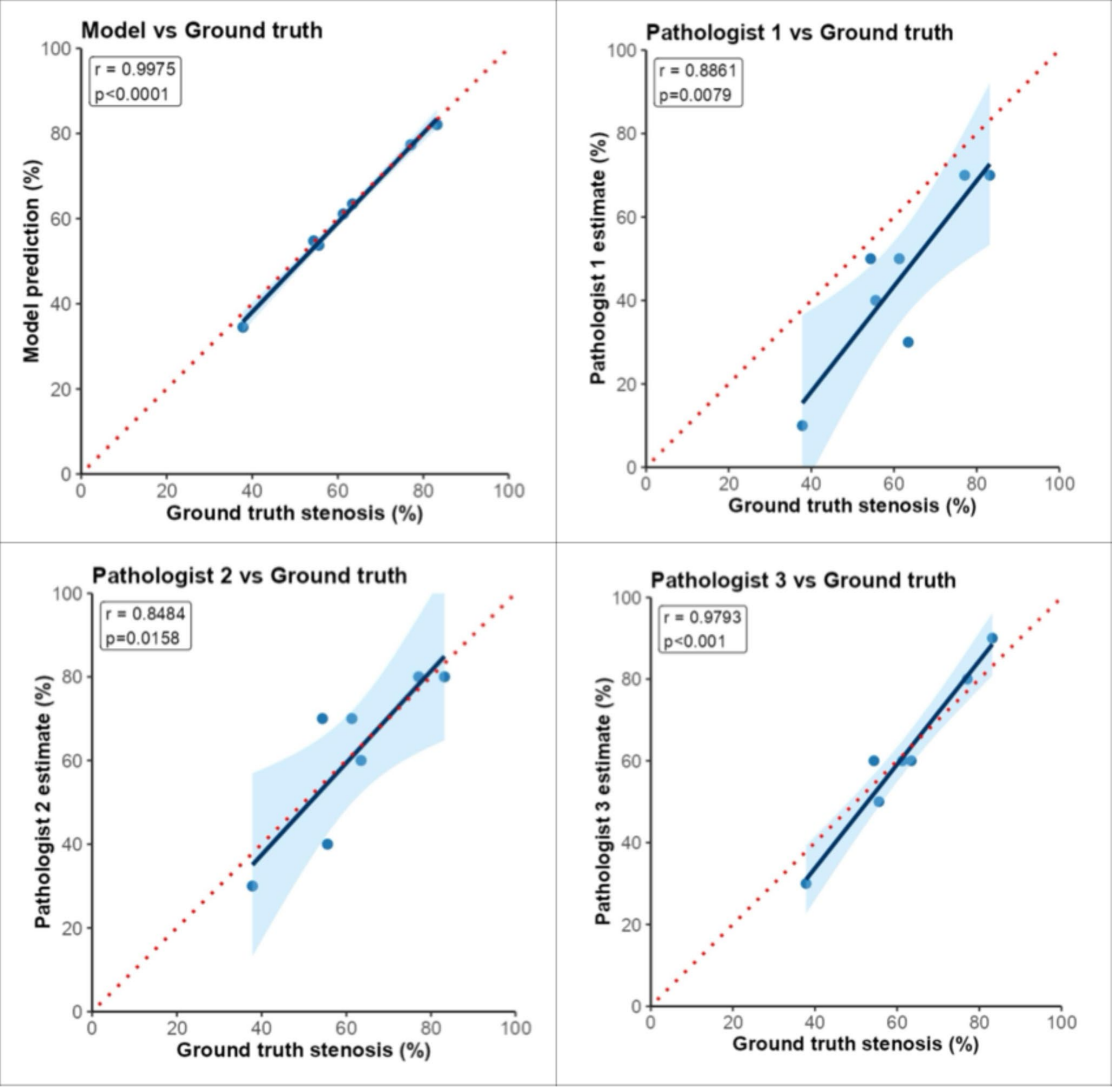
Scatterplots of ground-truth stenosis (%) versus model predictions and the three pathologists’ estimates. The solid line represents the fitted linear regression, the shaded area indicates the 95% confidence band, and the red dotted line denotes the line of identity (y = x). Pearson’s correlation coefficient (r) and the corresponding p-value are reported in each panel

In this stage, the model’s performance and the visual assessments of three independent forensic pathologists were compared to the ground truth. Results are shown in Table sm2 – supplementary material. Model and pathologists’ performance are reported in Tables sm3 and sm4 - supplementary material. Pearson correlation plots comparing each set of estimates with the ground-truth coronary stenosis are shown in Fig. 5.

Across the seven test ROIs (TD-1–TD-7), model predictions tracked the ground-truth stenosis closely, with model %Er ranging from − 8.75% (TD-1) to + 0.75% (TD-6), being notably 0.00% in TD-7. In contrast, the three pathologists showed larger and more variable deviations from ground truth at the ROI level: Pathologist 1 consistently underestimated stenosis (e.g., − 73.57% in TD-1 and − 52.73% in TD-7), whereas Pathologist 2 alternated between under- and overestimation (from − 28.01% in TD-3 to + 28.79% in TD-6), and Pathologist 3 showed smaller but still noticeable variability (from − 20.70% in TD-1 to + 10.40% in TD-6). In addition, these results support the hypothesis of poor interobserver reproducibility in the visual estimation of coronary artery stenosis.

Summary performance metrics confirmed these patterns (Table sm3 – supplementary material). Versus ground truth, the model had a mean %Er of − 1.80% (SD 3.35%, 95% CI [− 4.89%, + 1.29%]), with low absolute error (MAE 1.01 pp, RMSE 1.50 pp, MAPE 2.11%) and a very strong linear association (Pearson *r* = 0.9975, *p* < 0.0001, 95% CI [0.9823, 0.9996]). Pathologist 1 showed a markedly negative mean %Er (− 29.40%, SD 24.72%, 95% CI [− 52.26%, − 6.53%]) and substantially higher absolute error (MAE 16.11 pp, RMSE 18.92 pp, MAPE 29.40%), despite a positive correlation with ground truth (*r* = 0.8861, p 0.0079). Pathologist 2 had a near-zero mean %Er (− 1.60%) but high dispersion (SD 19.51%, 95% CI [− 19.65%, + 16.44%]) with MAE 8.18 pp, RMSE 9.67 pp, MAPE 14.96% (*r* = 0.8484, p 0.0158). Pathologist 3 showed intermediate performance (mean %Er − 2.27%, SD 10.92%, 95% CI [− 12.38%, + 7.83%]; MAE 4.79 pp, RMSE 5.25 pp, MAPE 8.66%) with high correlation (*r* = 0.9793, *p* < 0.001).

Bland–Altman analysis (Table sm4 – supplementary material) indicated the narrowest agreement band for the model (bias − 0.82 pp; 95% LoA [− 3.48, 1.83]) compared with the pathologists, with the widest LoA for Pathologist 2 ([− 20.85, 20.06]) and a large negative bias for Pathologist 1 (− 16.11 pp; LoA [− 37.13, 4.91]); bootstrap CIs for bias and LoA are reported in Table sm4 – supplementary material.

## Discussion

The quantification of coronary stenosis frequently constitutes a central phase in forensic autopsy. Nonetheless, traditional gross and histological visual assessments remain inherently susceptible to inter-observer variability, even among experienced forensic pathologists, who often rely on broad categorical indicators rather than on genuinely continuous measurements [8].

In routine autopsy practice, the designation of “critical” coronary stenosis is still commonly derived from macroscopic visual estimation performed by the forensic pathologist at the time of autopsy or, more recently, through WSIs analysis, which is rigorous but time-consuming [5, 25].

These issues are extremely relevant since major consensus documents for sudden cardiac death investigations explicitly use severe stenosis thresholds (commonly > 75% luminal stenosis) as supportive morphological evidence in appropriate contexts, making the numerical estimate potentially influential in causal attribution [5].

Standardization efforts, such as morphometrically generated visual guides, have been proposed to harmonize visual grading, but by design, they remain discretized (template-based steps) and cannot fully represent the continuous spectrum of eccentric and irregular plaques encountered in real cases [9].

From a medico-legal standpoint, this introduces significant inter-observer variability, potentially resulting in inconsistent stenosis severity classifications and, in borderline cases, conflicting interpretations of mechanism and causation. Indeed, in the forensic setting, WSI could provide the much-needed objectivity and should be regarded as more than a convenience: it creates a persistent digital file that can be securely archived, re-opened, and re-reviewed, enabling traceable and auditable handling of histological evidence in a manner that aligns with medico-legal expectations making possible standardization of histological evidence [26, 27].

In cardiovascular pathology specifically, digitalization enables a qualitative step-change from visual estimation of stenosis to explicit morphometry (areas and ratios measured on standardized images), allowing the quantitative endpoint to be tied to reproducible annotation and independently checked using stored overlays on the original WSI [15]. Nevertheless, this procedure is labor-intensive and time-consuming because it requires annotating ROIs each time a pathologist would like to assess vascular stenosis.

In this context, the development of an easy-to-use, rapid AI-assisted tool for estimating coronary stenosis appears helpful, time-saving, and essential.

In the present framework, deep learning model could provide objective measurements, even for continuous variable such as the percentage of stenosis, derived from pixel-wise segmentation of anatomically meaningful compartments (background, tunica media, tunica intima, and lumen), acting as a metrology layer that can support expert forensic interpretation.

Our developed model has been designed for application to forensic autopsy material, where tissue may be partially degraded or fragmented due to a relatively long post-mortem interval. To prevent the generation of biologically implausible outputs, the workflow incorporates anatomically constrained post-processing (e.g., lumen nested within the intima, and the intima within the media), applied only in instances where the model confidence is low. In addition, the model produces a hybrid confidence map that explicitly flags pixels modified during reconstruction, serving as a transparency or audit layer that the pathologist can review alongside the WSI.

This approach fits naturally within the field’s trajectory from discretized visual guides for stenosis estimation, to digital morphometry on WSI, and finally to deep learning for automation and scalability: visual templates (useful but inherently stepwise) have been complemented by open, reproducible digital pathology platforms and by forensic-oriented studies advocating quantitative digital analysis for coronary sub-occlusions, coming to the automatic segmentation and stenosis calculation [15].

Transformer-based segmentation backbones such as SegFormer provide an efficient architecture for semantic segmentation, while the explicit reporting of confidence or uncertainty aligns with a broader vision of deploying AI in high-stakes image analysis with interpretable uncertainty cues [21].

A rigorous labeling strategy is essential for developing a reliable model capable of generating standardized stenosis estimates. In the present context, the application of morphology-based annotations, performed in accordance with objective wall artery structures and supported by high-magnification assessment of the internal elastic lamina, grounds the training process in reproducible histological landmarks. Consequently, the objectivity of the model outputs depends not solely on algorithmic performance but also on the accuracy, consistency, and biological validity of the annotations utilized during training.

In our research study, we propose a digital pathology pipeline based on WSI morphometry, coupled with a deep-learning segmentation model, to provide rapid, auditable, continuous, and reproducible stenosis measurement that can be reviewed directly on the original archived WSI with traceable overlays.

Within the validation cohort (14 ROIs), model predictions were tightly aligned with the morphometry-derived ground truth: Pearson’s correlation was 0.986 (*p* < 0.001), indicating near-excellent agreement. On an absolute scale, the mean absolute error was 3.22 pp, and the mean percent error was − 2.87% (Table 2). These values support the internal consistency of the pipeline from ROI selection and mask generation (IEL and lumen) through segmentation-based area computation.

The most practically relevant signal emerges from the independent test set (7 ROIs), where the model maintained very low deviation from ground truth (MAE 1.01 pp). By contrast, pathologists’ estimates showed substantially larger errors (MAE 16.11 pp, 8.18 pp, and 4.79 pp for Pathologists 1–3, respectively) and wide dispersion at the single-ROI level (up to 30 pp between the highest and lowest estimates for the same ROI).

Agreement analyses consistently favored the model over visual estimation. Bland–Altman results showed that the model had a small bias versus ground truth (− 0.82 pp) and tight 95% limits of agreement (− 3.48 to 1.83 pp), indicating minimal systematic deviation and low random error across ROIs. In contrast, visual estimates exhibited substantially wider agreement bands, reflecting both greater dispersion and, for some observers, systematic underestimation. Concordantly, the model achieved the highest absolute agreement (0.9953) compared with the pathologists (0.6159–0.9544), supporting its superior reliability compared with the visual coronary stenosis assessment method.

In other words, while the human estimates drifted considerably and inconsistently from the ground truth, the model remained close to the morphometric reference (i.e., the ground truth) across all the WSIs. Taken together, these findings suggest that the proposed AI tool can function as an objective, continuous-output “measurement layer” that mitigates the known limitations of solely categorical visual grading.

The practical applicability of the proposed tool relies on appropriate coronary artery sampling during autopsies, with histological slides obtained from adequately selected transverse sections and, when necessary, after decalcification, so as to preserve interpretable vessel wall and luminal morphology for reliable stenosis estimation.

In addition, rapid processing time (≈ 28.44 s per image) underscores the efficiency of the AI workflow, enabling timely assessment of coronary stenosis with minimal computational delay.

The forensic value of an AI-assisted measurement extends beyond mere numerical precision and processing speed; it also depends on whether the method can be articulated, tested, and audited in accordance with legal standards for expert witness evidence, the ultimate goal of medico-legal investigations. Under Daubert, courts emphasize testability, peer review, known or knowable error rates, standards controlling technique operation, and (where relevant) general acceptance [28].

Our experimental approach was designed to align with such standards. First, it is explicitly testable: performance can be re-evaluated prospectively on new cases and independently audited using stored WSIs and corresponding masks. Second, it yields a known and measurable error profile (Tables 2, sm1, sm2, sm3 and sm4 - supplementary material), which can be updated as external validations accumulate. Third, the method is standardized operationally (scanner settings, ROI criteria, confidence thresholds, post-processing steps), with outputs preserved as overlays linked to the original image data, features that directly support auditability under legal standards.

Importantly, within a medico-legal context, the model should be framed as a decision support tool: the definitive conclusion regarding the cause of death remains a synthesis that incorporates gross findings, histology, toxicology, circumstances, and competing mechanisms.

Beyond the cause of death identification, in the context of medico-legal investigation involving potential medical malpractice related to coronary artery disease, accurately determining the percentage of coronary stenosis plays a central role in evaluating whether revascularization procedures, such as percutaneous coronary intervention or bypass surgery, would have been clinically indicated to prevent adverse outcomes like myocardial infarction or sudden death. Indeed, according to the 2021 ACC/AHA/SCAI Guideline for Coronary Artery Revascularization, stenoses are deemed hemodynamically significant and often warrant revascularization at specific thresholds [29].

Although histopathology cannot be regarded as a direct surrogate for in vivo coronary imaging, the well-documented clinicopathological correlation for evaluating coronary stenosis supports the use of standardized postmortem histomorphometric quantification as an objective benchmark in retrospective forensic analysis [30]. In this regard, our segmentation model may facilitate the assessment of disputed cases by offering a reproducible ex vivo estimation of luminal narrowing, which should be interpreted in conjunction with clinical imaging results and with prudence in complex, severe, or distal lesions.

Several limitations deserve emphasis. While the overall dataset is non-trivial for pixel-wise annotation (103 ROIs), the test set is small and derived from two Forensic Medicine Institutes. External validation across institutions, different scanners, staining variability, and broader lesion morphologies is required before any generalization. In addition, ROIs were selected for high quality; real-world forensic material may include more artefacts (folds, tearing, autolysis, decalcification effects) that could degrade segmentation performance. Finally, the ground truth relies on mask fidelity; residual annotation uncertainty and borderline pixels at the IEL interface can propagate to the final stenosis estimate.

Overall, our findings support the feasibility of an AI-assisted, WSI-based quantitative approach to coronary stenosis assessment in forensic histopathology. The model demonstrated minimal error relative to a morphometric reference and markedly reduced variability compared with unaided visual estimates, improving time efficiency.

## Conclusion

We developed and internally validated a WSI-based deep learning tool to support objective quantification of coronary artery stenosis in forensic autopsies, a setting in which conventional visual estimation remains vulnerable to inter-observer variability and can influence causal attribution in sudden cardiac death investigations. By coupling a standard digital pathology infrastructure (WSI acquisition and secure archiving) with transformer-based segmentation, our approach enables continuous, traceable measurement. In addition, integrating a hybrid confidence map improves biological plausibility and provides an auditable transparency layer for the end user.

Against the morphometric ground truth, the AI-driven tool predictions showed a small overall bias and low absolute error, with a strong correlation with the reference. Moreover, in a blinded test, the model showed minimal deviation from ground truth and demonstrated substantially greater consistency compared to independent visual estimates by experienced pathologists.

These data highlight the value of adopting an AI-driven tool applied to WSIs to ensure accurate and time-efficient assessment of coronary stenosis in post-mortem specimens. Importantly, quantification of coronary stenosis must remain an integral part of a comprehensive autopsy synthesis that integrates gross findings, histology, toxicology, and relevant circumstances, when available.

## Supplementary Information

Below is the link to the electronic supplementary material.

Supplementary Material 1

## Data Availability

Data supporting the findings of this study are available from the corresponding author upon reasonable request, subject to legal and institutional restrictions.
