# Supplementary material for: Development of a deep learning-based tool for coronary artery stenosis evaluation in forensic autopsies using whole slide imaging
Source: Int J Legal Med. 2026 Apr 18;140(4):2113–25. doi: 10.1007/s00414-026-03800-6 (PMC13275746; doi:10.1007/s00414-026-03800-6)
Supplement: Supplementary file 1 — Supplementary Material 1 [file 414_2026_3800_MOESM1_ESM.docx]

| ***ROI ID*** | ***Ground Truth Stenosis*** | ***Model Stenosis Prediction*** | ***Model Prediction Er.*** | ***Model Prediction %Er.*** |
| --- | --- | --- | --- | --- |
| ***VD-1*** | 6.12% | 5.72% | -0.40 pp | -6.54% |
| ***VD-2*** | 42.64% | 36.09% | -6.55 pp | -15.36% |
| ***VD-3*** | 57.82% | 54.62% | -3.20 pp | -5.53% |
| ***VD-4*** | 62.75% | 64.30% | 1.55 pp | 2.47% |
| ***VD-5*** | 55.42% | 54.87% | -0.55 pp | -0.99% |
| ***VD-6*** | 93.96% | 92.71% | -1.25 pp | -1.33% |
| ***VD-7*** | 51.49% | 54.04% | 2.55 pp | 4.95% |
| ***VD-8*** | 46.42% | 44.05% | -2.37 pp | -5.11% |
| ***VD-9*** | 65.16% | 58.66% | -6.50 pp | -9.98% |
| ***VD-10*** | 62.46% | 58.17% | -4.29 pp | -6.87% |
| ***VD-11*** | 52.26% | 48.34% | -3.92 pp | -7.50% |
| ***VD-12*** | 57.67% | 65.95% | 8.28 pp | 14.35% |
| ***VD-13*** | 97.33% | 97.93% | 0.60 pp | 0.62% |
| ***VD-14*** | 91.13% | 88.11% | -3.02 pp | -3.31% |

**Table sm1.** Results of validation dataset (VD = validation dataset; Er = percentage points error; %Er = percent error; pp = percentage points).

| ***ROI ID*** | ***Ground Truth*** | ***Model Prediction*** | ***Pathologist 1***  ***Visual Est.*** | ***Pathologist 2***  ***Visual Est.*** | ***Pathologist 3***  ***Visual Est.*** | ***Model Prediction %Er*** | ***Pathologist 1 Visual Est %Er*** | ***Pathologist 2***  ***Visual Est***  ***%Er*** | ***Pathologist 3***  ***Visual Est***  ***%Er*** |
| --- | --- | --- | --- | --- | --- | --- | --- | --- | --- |
| ***TD-1*** | **37.83%** | 34.52% | 10.00% | 30.00% | 30.00% | -8.75% | -73.57% | -20.70% | -20.70% |
| ***TD-2*** | **61.29%** | 61.09% | 50.00% | 70.00% | 60.00% | -0.33% | -18.42% | 14.21% | -2.10% |
| ***TD-3*** | **55.56%** | 53.74% | 40.00% | 40.00% | 50.00% | -3.28% | -28.01% | -28.01% | -10.01% |
| ***TD-4*** | **83.18%** | 82.08% | 70.00% | 80.00% | 90.00% | -1.32% | -15.85% | -3.82% | 8.20% |
| ***TD-5*** | **77.10%** | 77.35% | 70.00% | 80.00% | 80.00% | 0.32% | -9.21% | 3.76% | 3.76% |
| ***TD-6*** | **54.35%** | 54.76% | 50.00% | 70.00% | 60.00% | 0.75% | -8.00% | 28.79% | 10.40% |
| ***TD-7*** | **63.46%** | 63.46% | 30.00% | 60.00% | 60.00% | 0.00% | -52.73% | -5.45% | -5.45% |

**Table sm2**. Results of test dataset (Est.: estimation; TD: test dataset; %Er = percentage error).

| ***Comparison vs GT*** | ***Mean %Er*** | ***SD*** | ***95% CI of mean %Er*** | ***MAPE %*** | ***MAE (pp)*** | ***RMSE (pp)*** | ***Pearson correlation (r)*** | ***p (two-tailed)*** | ***95% CI*** |
| --- | --- | --- | --- | --- | --- | --- | --- | --- | --- |
| ***Model prediction*** | **−1.80%** | 3.35% | **[−4.89%, +1.29%]** | 2.11 | 1.01 | 1.50 | **0.9975** | **<0.0001** | **[0.9823, 0.9996]** |
| ***Pathologist Visual Est. 1*** | **−29.40%** | 24.72% | **[−52.26%, −6.53%]** | 29.40 | 16.11 | 18.92 | **0.8861** | **0.0079** | **[0.3999, 0.9831]** |
| ***Pathologist Visual Est. 2*** | **−1.60%** | 19.51% | **[−19.65%, +16.44%]** | 14.96 | 8.18 | 9.67 | **0.8484** | **0.0158** | **[0.2642, 0.9772]** |
| ***Pathologist Visual Est. 3*** | **−2.27%** | 10.92% | **[−12.38%, +7.83%]** | 8.66 | 4.79 | 5.25 | **0.9793** | **<0.001** | **[0.8618, 0.9971]** |

**Table sm3**. Model and pathologists’ performance on the test dataset. (GT = ground truth; %Er = percent error; SD = standard deviation CI = confidence interval; MAPE = mean absolute percentage error; MAE = mean absolute error; pp = percentage points; RMSE = root mean square error; Est.: estimation).

| ***Comparison vs GT*** | ***Bland–Altman bias (pp)*** | ***95% LoA (pp)*** | ***Bias 95% CI (boot)*** | ***LoA low 95% CI (boot)*** | ***LoA high 95% CI (boot)*** | ***ICC*** | ***ICC 95% (boot)*** |
| --- | --- | --- | --- | --- | --- | --- | --- |
| ***Model prediction*** | **−0.82** | **[−3.48, 1.83]** | [−1.83, 0.00] | [−4.98, −1.02] | [0.54, 2.41] | 0.995 | [0.980, 0.999] |
| ***Pathologist Visual Est. 1*** | **−16.11** | **[−37.13, 4.91]** | [−23.78, −9.33] | [−46.28, −17.00] | [−5.57, 9.80] | 0.616 | [0.161, 0.785] |
| ***Pathologist Visual Est. 2*** | **−0.40** | **[−20.85, 20.06]** | [−7.45, 6.83] | [−28.54, −8.12] | [4.73, 28.48] | 0.842 | [0.309, 0.956] |
| ***Pathologist Visual Est. 3*** | **−0.40** | **[−11.47, 10.68]** | [−4.16, 3.44] | [−14.72, −4.59] | [2.92, 14.06] | 0.954 | [0.821, 0.967] |

**Table sm4**. Model and pathologists’ performance on the test dataset. (GT = ground truth; LoA = level of agreement; Est.: estimation)
